# Peer Victimization and Problematic Online Game Use Among Chinese Adolescents: The Dual Mediating Effect of Deviant Peer Affiliation and School Connectedness

**DOI:** 10.3389/fpsyg.2022.823762

**Published:** 2022-03-22

**Authors:** Hao Li, Xiong Gan, Guo-Xing Xiang, Ting Zhou, Pinyi Wang, Xin Jin, Congshu Zhu

**Affiliations:** ^1^Department of Psychology, College of Education and Sports Sciences, Yangtze University, Jingzhou, China; ^2^Department of Psychology, College of Education and Sports Sciences, Yangtze University College of Technology and Engineering, Jingzhou, China

**Keywords:** peer victimization, problematic online game use, deviant peer affiliation, school connectedness, adolescence

## Abstract

Abundant evidence has proved an association between peer victimization and problematic online game use (POGU). However, the underlying mechanisms of this relation are still under-investigated. Grounded in the ecological system theory, this study examined whether deviant peer affiliation (DPA) and school connectedness mediated the association between peer victimization and adolescent POGU. A sample of 698 Chinese adolescents completed questionnaires regarding peer victimization, problematic online game use, DPA, and school connectedness, of which 51.58% were boys. Path analyses indicated that peer victimization was positively associated with problematic online game use, and this link could be mediated by deviant peer affiliation and school connectedness. The findings identify the potential underlying mechanism by which peer victimization is associated with adolescent problematic online game use, which has important implications for theory and prevention.

## Introduction

As of June 2021, the number of Internet users in China has reached 1 billion, while the number of online game users has reached 510 million, including more than 100 million young users (CINIC, 2021). Whereas low to moderate levels of online gaming may be entertaining and provide opportunities to interact with other players online, excessive gaming can lead to problematic online game use (POGU) and seriously damage the physical and mental health, personality development, academic adaptation, interpersonal relationships, and other social functions of adolescents (Griffiths and Nuyens, 2017; Wang et al., 2020b). POGU is a subtype of problematic internet use and is defined as the “uncontrollable, excessive, and compulsive use of online games that causes serious problems such as social and/or emotional problems” (Young and Abreu, 2011). As POGU has become a global public health issue, it has been included in the updated version of the Diagnostic and Statistical Manual Disorders (DSM-5) and the International Classification of Diseases (ICD-11) and is thus attracting enormous attention from researchers (Arbanas, 2015; WHO, 2018). However, the research in this field has not been fully tested to explore when, where, and how to influence adolescent problematic online game use. In order to develop more effective prevention strategies, it is imperative to examine potential risk factors and mechanisms of Internet addiction among adolescents.

The ecological system theory (Bronfenbrenner, 1977) points out that the development of adolescents will be affected by multiple ecological subsystems such as peers [peer victimization or deviant peer affiliation (DPA)] and school (school connectedness). In recent years, among the many influencing factors of adolescent POGU, the role of peer victimization has received more and more researchers’ attention (Xu and Chang, 2003; Li and Zhu, 2020; Liang et al., 2021). Peer victimization refers to the experience of peer aggression or bullying, which is a salient stressor for adolescents (Salmivalli and Peets, 2009). As we all know, peer relations are vitally important for adolescence, and a good peer relationship can buffer an adolescent from adverse effects (Hoorn et al., 2016). In contrast, peer victimization can seriously endanger the physical and mental health of an adolescent (Farrell and Vaillancourt, 2019; Spiekerman et al., 2020). Previous studies have demonstrated that peer victimization is closely related to adolescent POGU (Su et al., 2018; Wang et al., 2020a). Experiencing peer victimization may lead to adolescents adopting maladaptive coping strategies, such as relying on online gaming to escape stressful situations and relieve negative emotions (Hsieh et al., 2019). Thus, it is reasonable to assume that peer victimization is a particularly critical risk factor for adolescent POGU.

In order to maximize the effectiveness of interventions to prevent the negative effects of POGU, our goal in the current study is to explore the mediators that underlie this relationship. Specifically, we examined whether DPA and school connectedness mediate the relationship between peer victimization and adolescent POGU using the ecological system theory.

### Deviant Peer Affiliation as a Mediator

However, the relationship between peer victimization and problematic online game use seems to be mediated by other variables (Zhai et al., 2019). DPA refers to adolescents making friends with peers who are involved in deviant behaviors such as cheating, fighting, substance abuse, and indulge in online games (Wang et al., 2017). Victimized adolescents tend to reduce engagement with their mainstream peer groups due to peer rejection and increase their association with deviant peers (Jiang et al., 2016). According to social network theory, adolescents are eager to build friendships with their peers and join peer groups. Victimized adolescents are more likely to select deviant peers who are perceived similarly as social outcasts as friends when they are isolated from mainstream groups (Lazarsfeld and Merton, 1954; Veenstra and Dijkstra, 2014). Indeed, recent studies have found that peer victimization was positively correlated with DPA and increased the possibility of DPA (Rudolph et al., 2014; Su et al., 2018).

Moreover, individuals can learn similar behaviors by observing and mimicking their peers’ negative behaviors (such as indulging in online games; Bandura, 1977). The deviance regulation theory suggests that group attitudes and norms formed by deviant peers will exert pressure on adolescent behavior. Only by maintaining negative behaviors (such as indulging in online game) can individuals gain reinforcement from the group and avoid punishment caused by conflicts with group norms (Blanton and Christie, 2003). Empirical studies have identified significant associations between DPA and POGU in adolescents (Zhu et al., 2015; Li et al., 2020). In addition, Chen et al. (2020) have also found that bullied teenagers can deepen their problematic behaviors through deviant peer relationships. Therefore, we hypothesize that DPA will mediate the association between peer victimization and adolescent POGU.

### School Connectedness as a Mediator

School connectedness is defined as “students’ perceptions that adults care about their learning and about them as individuals” (Fredricks, 2004). According to the social control theory (Hirschi and Travis, 1969), adolescents with lower levels of school connectedness are less likely to form an attachment to school or teachers, and they will fail to conform to the values, norms, and beliefs of the school, and for this reason, display more problematic behaviors such as POGU. Several empirical studies have found that low school connectedness is related to increased adolescent POGU (Zhu et al., 2015; Liu et al., 2017; Tian et al., 2018). For instance, Zhu et al. (2015) conducted a longitudinal study on 876 Chinese adolescents, and revealed that diminished school connectedness increased the risk of adolescent POGU.

Furthermore, peer victimization may predict adolescents’ low school connectedness. When adolescents are bullied by their peers, they may become less engaged in school activities because they perceive fewer emotionally supportive, warm, and positive interactions at school (Halliday et al., 2021). Previous research has found that peer victimization is negatively correlated with school connectedness (Wang et al., 2015; Dorio et al., 2019). In a longitudinal study, Ladd et al. (2017) found that peer victimization can predict adolescents’ school participation and high-chronic victimization was consistently associated with lower school connectedness, academic self-perceptions, and academic achievement. And the latest research also demonstrates that teenagers injured by peers can affect their physical and mental health through school connectedness (Eugene et al., 2021). Hence, we hypothesize that school connectedness will mediate the association between peer victimization and adolescent POGU.

### The Present Study

To sum up, most current studies pay more attention to the relationship between peer victimization, DPA, or school connectedness as a single factor and adolescent POGU, while few studies combine these four factors to consider the relationship. Under the basic framework of ecological system theory, the current study aims to bring together social network theory, deviance regulation theory, and social control theory to hypothesize that DPA and school connectedness will mediate the association between peer victimization and adolescent POGU.

## Materials and Methods

### Participants and Procedures

The participants in this study were recruited from two junior middle schools in Hubei, China through random cluster sampling. Our survey was conducted on a class basis, and a total of 698 adolescents (51.58% male) ranging in age from 12 to 16 (M_age_ = 13.52, SD = 0.70) participated in this study (effective recovery 94.32%). Moreover, the eligible participants were selected based on the following criteria: (1) participants who were adolescents, (2) adolescents who received consent from their guardians to participate, and (3) adolescents who agreed to participate. The present study was approved by the Research Ethics Committee of the College of Education and Sports Sciences, Yangtze University. Participants and their parents or legal guardians were provided with written consent forms, which informed them that personal information would be kept confidential and their responses would be used only for research purposes. The data was collected by trained psychology teachers or graduate students in psychology. To encourage honest reporting, adolescents were given approximately 30 min to complete the anonymous questionnaires.

### Measures

#### Peer Victimization

Peer victimization was measured by the Peer Victimization Questionnaire (Yu et al., 2017). The Peer Victimization Questionnaire is a self-report scale that is designed for Chinese adolescents, that includes nine activities that were measured for physical, verbal, and relational victimization. Example activities include “Have you ever been insulted by a companion” and “Have you been deliberately beaten, kicked, pushed, or bumped by a companion.” Respondents were asked to indicate the number of times that they had engaged in specific peer victimization activities in the last 6 months on a three-point scale (1 = never, 3 = more than or equal to three times). Mean scores were calculated, with higher scores indicating greater severity of peer victimization. This measure demonstrated good reliability and validity among Chinese adolescents (Yu et al., 2017). In the present study, the Cronbach’s alpha was 0.86.

#### Deviant Peer Affiliation

Deviant peer affiliation was measured with the 12-item self-report questionnaires (Zhu et al., 2015). The participants were asked to indicate the frequency with which their friends had participated in 12 kinds of deviant behaviors such as fighting, truancy, stealing, smoking, and cheating on exams during the past 6 months. All items were rated on a five-point scale ranging from 1 (never) to 5 (six or more). Mean scores were calculated, with higher scores meaning a greater level of DPA. This measure demonstrated good reliability and validity among Chinese adolescents (Lin et al., 2020). In the present study, the Cronbach’s alpha was 0.89.

#### School Connectedness

School connectedness was measured with the eight-item self-report questionnaire (Fredricks et al., 2004). The participants were asked to indicate the frequency of their school connectedness during the past half year. Example items include “I am happy and safe to be at this school.” All items were rated on a five-point scale ranging from 1 (never) to 5 (frequently recurring). Mean scores were calculated, with higher scores meaning higher levels of school connectedness. This measure demonstrated good reliability and validity among Chinese adolescents (Wang et al., 2011). In the present study, the Cronbach’s alpha was 0.74.

#### Problematic Online Game Use

Problematic online game use was measured with the 11-item Chinese version self-report questionnaire (Yu et al., 2017), which was adapted from the pathological-gaming scale (Gentile, 2009). The participants were asked to report the frequency of 11 symptoms of POGU over the past 6 months, such as “Do you need to spend more and more time on online games to be satisfied?” and “Do you become restless or irritable when attempting to cut down or stop playing online games?.” All items were rated on a three-point scale (from 0 = never to 2 = frequently). Mean scores were calculated, with higher scores meaning a higher risk of POGU, and the cut-off score for identifying adolescents with POGU was 5 or more (Young, 2011). This measure demonstrated good reliability and validity among Chinese adolescents (Lin et al., 2020; Liang et al., 2021). In the present study, the Cronbach’s alpha was 0.82.

### Statistical Analysis

Full information maximum likelihood (FIML) was used to estimate missing data. Descriptive and Pearson correlations statistical analyses were conducted with SPSS 24.0. And according to previous studies, mediation effects were tested through structural equation modeling using Mplus 7.4 (Wen et al., 2004b; Muthén and Muthén, 2011). Moreover, we adopted maximum likelihood estimation and bootstrapping with 2,000 replicates to test the hypothesis model.

## Results

### Preliminary Analyses

Considering that the self-report method may have common method bias, the common method bias was tested by Harman’s single factor test method (Podsakoff et al., 2012). The unrotated principal component factor analysis found that there are eight common factors with a characteristic root greater than 1, and the interpretation rate of the first factor is 20.97%, less than the critical standard of 40%. Therefore, the common method deviation of this study is not serious.

Means, SDs, and bivariate associations are shown in Table 1. As can be seen in the table, peer victimization was positively correlated with DPA (*r* = 0.36, *p* < 0.001) and POGU (*r* = 0.26, *p* < 0.001). DPA was positively associated with POGU (*r* = 0.34, *p* < 0.001). School connectedness was negatively associated with peer victimization (*r* = −0.23, *p* < 0.001), DPA (*r* = −0.19, *p* < 0.001), and POGU (*r* = −0.26, *p* < 0.001).

**Table 1:** Descriptive statistics and intercorrelations between variables.

Variable	*M*	*SD*	1	2	3	4
1. Peer victimization	1.60	0.47	1.00			
2. Deviant peer affiliation	1.68	0.73	0.36^**^	1.00		
3. School connectedness	2.40	0.72	−0.23^**^	−0.19^**^	1.00	
4. Problematic online game use	0.44	0.36	0.26^**^	0.34^**^	−0.26^**^	1.00

** *p < 0.01*.

The independent sample *t*-test was used to detect gender and grade differences in the POGU among adolescents. Results showed that POGU of boys was significantly higher than that of girls (*M*_boys_ = 0.57, *M*_girls_ = 0.30, *t* = 10.92, *p* < 0.001). But there were no significant grade differences (*t* = −0.22, *p* > 0.05).

### Mediational Analyses

To explore the mediation effects of DPA and school connectedness, a full model was established prior to examining the structural components of each model. The models consisted of four latent variables: peer victimization, DPA, school connectedness, and POGU. Since DPA, school connectedness, and POGU scales are unidimensional measures, all items were parceled for each scale to be used as observation indicators by the item-to-construct balance method (Little et al., 2002), which was in order to keep the estimation stability of the models and reliability of data fitting, and to reduce the data deviation. Peer victimization includes the observed variables of physical, verbal, and relational victimization. The maximum likelihood estimation method is used to estimate and test the parameters of the measurement model. Results showed that the measurement model fit the data well (Wen et al., 2004a; *χ*^2^/*df* = 2.19, *CFI* = 0.99, *TLI* = 0.98, *RMSEA* = 0.04, and *SRMR* = 0.03).

**Figure 1:**
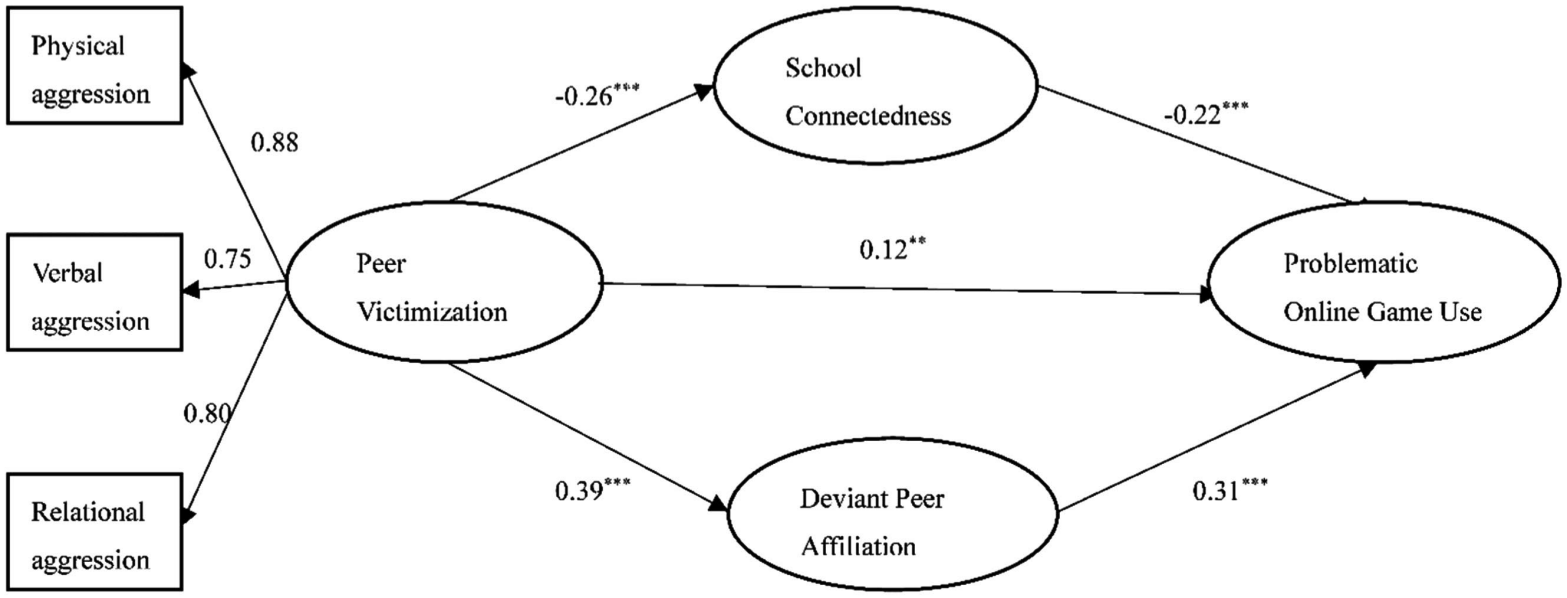
Path model results with standardized coefficients (*n* = 698). ^***^p < 0.001, ^**^p < 0.01.

We tested the mediation effects of DPA and school connectedness by following several steps. First, the direct effect of peer victimization on adolescent POGU was tested, with peer victimization as the predictive variable and POGU as the outcome variable. The model had a good fit to the data (*χ*^2^/*df* = 3.10, *CFI* = 0.99, *TLI* = 0.98, *RMSEA* = 0.06, and *SRMR* = 0.03). The results revealed that the direct path from peer victimization to POGU was significant (*β* = 0.29, *p* < 0.001). Then we added two mediation variables, DPA and school connectedness, to the model. The mediation model fit the data well (*χ*^2^/*df* = 2.33, *CFI* = 0.98, *TLI* = 0.98, *RMSEA* = 0.04, and *SRMR* = 0.04). The results are presented in Figure 1, which indicates that all paths were statistically significant.

Bootstrapping analyses was used to test the relationship between each path. The results indicated that DPA had a significant positively indirect effect in the association between peer victimization and POGU [indirect effect = 0.12, 95% CI (0.07, 0.18)], while school connectedness had a significant negatively indirect effect in the association between peer victimization and POGU [indirect effect = 0.06, 95% CI (0.03, 1.00)]. The mediation effect accounts for 62.07% of the total effect.

## Discussion

Although some empirical has shown that there is a positive association between peer victimization and adolescent POGU, the mediating mechanisms underlying this relationship have not been identified. According to ecological system theory (Bronfenbrenner, 1977), social network theory (Lazarsfeld and Merton, 1954), deviance regulation theory (Blanton and Christie, 2003), social control theory (Hirschi and Travis, 1969), and empirical research, the current study examined the mediation effect of DPA and school connectedness on the relationship between peer victimization and adolescent POGU. Findings indicated that peer victimization has a positive effect on adolescent POGU, and the relationship between peer victimization and POGU was mediated by DPA and school connectedness. These observations expand our understanding of the complex relations between peer victimization and POGU among teenagers in China and provide reference suggestions for the prevention and intervention of POGU in adolescents.

The present study indicated that peer victimization was positively correlated with POGU, which was consistent with previous findings (Jia et al., 2018; Su et al., 2018; Wang et al., 2020a). It showed that peer victimization was an important risk predictor of adolescent POGU, that is, the more peer victimization, the greater the risk of adolescent POGU. Victimized adolescents are often rejected by other peer groups, and are prone to feeling lonely and negative emotions due to a lack of close partnership (Juvonen and Graham, 2014; Jetelina et al., 2019). The Internet provides a wider and more diversified space, which makes them more likely to use the Internet and play online games to escape from their real-life environment and avoid negative emotions (Elhai et al., 2017). This in turn increases the risk of overuse of adolescent Internet gaming.

Moreover, structural equation model analysis indicated that the mediating effect of DPA and school connectedness was significant, that is, peer victimization can not only directly predict POGU, but also indirectly affect POGU through DPA and school connectedness. The ecological system theory indicates that the development of adolescents will be affected by multiple ecological subsystems such as peers and school (Bronfenbrenner, 1977). On the one hand, peer victimization is positively associated with DPA, which is a peer ecological subsystem variable. This finding is consistent with prior studies (Rudolph et al., 2014; Su et al., 2018). According to social network theory (Lazarsfeld and Merton, 1954; Veenstra and Dijkstra, 2014), adolescents value peer relationships and desire to establish friendships, but due to the rejection of mainstream peer groups, victimized adolescents may choose to associate with deviant peers. At the same time, consistent with previous research (Zhu et al., 2015; Li et al., 2020), DPA is positively associated with adolescent POGU. Social learning theory (Bandura, 1977) indicates that individuals can indulge in online games by observing and imitating their peers’ negative behaviors. Deviance regulation theory (Blanton and Christie, 2003) also reveals that victimized adolescents have to imitate and maintain deviant behaviors (such as indulging in online games) in the process of interacting with deviant peers, and gradually form positive views on problem behaviors, so as to develop deviant behaviors. Finally, this finding further illustrates that not only cyber bullying (Chen et al., 2020) but also peer victimization in real-life can promote communication with deviant peers, which leads to problem behavior. According to this, educators should pay attention to the impact of DPA on adolescent POGU, be alert to the phenomenon of deviant peer gatherings, and guide teenagers to stay away from deviant companions and avoid problematic behavior (Zhu et al., 2015; Li et al., 2020).

On the other hand, school connectedness, as a school ecosystem subsystem variable, can also mediate peer victimization and POGU. First of all, peer victimization is negatively associated with school connectedness, which is consistent with previous studies (Wang et al., 2015; Dorio et al., 2019). It will be difficult for victimized adolescents to build trust with teachers and classmates, and due to less emotional support, warmth, and positive interaction in school, their level of school connectedness is low (Buhs et al., 2006; Frojd et al., 2008; Garvik et al., 2014). Additionally, school connectedness is negatively associated with POGU and this finding is consistent with previous research (Zhu et al., 2015; Liu et al., 2017; Tian et al., 2018). According to the social control theory (Hirschi and Travis, 1969), adolescents with a lower level of school connectedness are less likely to conform to the values, norms, and beliefs of the school, and therefore exhibit more problematic behaviors such as POGU (Wang and Fredricks, 2014). In addition, the results confirm that teenagers who are bullied by their peers will weaken their connection with the school, leading to internalization problems (Eugene et al., 2021) or problem behavior (such as problematic online game use). And once again verified the applicability of the ecosystem theory. Therefore, educators can strengthen interaction and communication with students by carrying out high-quality school activities in order to help students obtain interpersonal support from teachers and other peers, to improve students’ sense of connectedness to school, and to reduce their level of problematic online game use (Wang and Fredricks, 2014; Xie et al., 2017).

## Strengths, Limitations, and Future Directions

There are a few advantages to this study that should be mentioned. Firstly, the sample size is large, with nearly 700 middle school students participating in the survey. Secondly, the theory is sufficient. According to ecological system theory, social network theory, deviance regulation theory, and social control theory, the current study discovered the mediation effect of DPA and school connectedness on the relationship between peer victimization and adolescent POGU. On the one hand, the research conclusions further deepen the previous studies and once again verify the applicability of the ecosystem theory. On the other hand, our findings suggest that it is important to strengthen school connectedness and improve peer relationships so as to reduce the possibility of problematic online game use.

The limitations of this study and future directions should be noted. First, this study used a cross-sectional research design. Therefore, the causal relationships cannot be inferred. Future longitudinal or experimental studies can further determine the direction of the effects. Second, the data were collected only through self-reported measures. Self-reports may be subject to increased biases (e.g., socially desirable responses) and inflated associations between antecedents and outcome variables (Podsakoff et al., 2012). Reports from multiple informants (e.g., parents, teachers, and peers) should be considered in future research. Third, the sample was limited to adolescents drawn from two junior middle schools in China. Thus, caution is warranted in generalizing the findings to other cultures. Finally, the results of the present study also need to be extended to a more representative sample of Chinese adolescents and adolescents from other cultural backgrounds for a wider test.

## Conclusion

Peer victimization can be positively associated with adolescent POGU, and DPA and school connectedness play a parallel mediating role between peer victimization and POGU among adolescents. Educators can strengthen interaction and communication with students to help students obtain interpersonal support from teachers and other peers, thereby reducing their level of problematic online game use.

## Data Availability Statement

The raw data supporting the conclusions of this article will be made available by the authors, without undue reservation.

## Ethics Statement

The studies involving human participants were reviewed and approved by the Research Ethics Committee of the College of Education and Sports Sciences, Yangtze University. Written informed consent to participate in this study was provided by the participants’ legal guardian/next of kin.

## Author Contributions

HL, XG, and TZ designed the work. XG, PW, and XJ collected the data. HL, PW, and CZ analyzed the data results and drafted the manuscript. HL, G-XX, XG, TZ, XJ, and CZ revised the manuscript. All authors contributed to the article and approved the submitted version.

## Funding

The present study was funded by Youth Project of Natural Science Foundation of Hubei Province in 2020 (2020CFB365) and Achievements of Key Projects of Education Science Plan of Hubei Province in 2019 (2019GA017).

## Conflict of Interest

The authors declare that the research was conducted in the absence of any commercial or financial relationships that could be construed as a potential conflict of interest.

## Publisher’s Note

All claims expressed in this article are solely those of the authors and do not necessarily represent those of their affiliated organizations, or those of the publisher, the editors and the reviewers. Any product that may be evaluated in this article, or claim that may be made by its manufacturer, is not guaranteed or endorsed by the publisher.
